# Sarcopenia—A Valuable Imaging Biomarker for Disease Progression in Patients With Primary Sclerosing Cholangitis (PSC)?

**DOI:** 10.1111/liv.70328

**Published:** 2025-09-09

**Authors:** Alena Levers, Judith Pantke, Filip Klimeš, Henrike Lenzen, Daniel Düx, Richard Taubert, Heiner Wedemeyer, Frank Wacker, Kristina I. Ringe

**Affiliations:** ^1^ Hannover Medical School Department of Diagnostic and Interventional Radiology Hannover Germany; ^2^ European Reference Network on Hepatological Diseases (ERN RARE‐LIVER) Hamburg Germany; ^3^ Department of Gastroenterology, Hepatology, Infectious Diseases and Endocrinology Hannover Medical School Hannover Germany

**Keywords:** biomarker, MRI, primary sclerosing cholangitis, sarcopenia

## Abstract

**Background and Aims:**

We aimed to ascertain the prevalence of sarcopenia in patients with primary sclerosing cholangitis (PSC) and to assess the prognostic value as a biomarker for disease outcome.

**Methods:**

We collected data from 224 patients (148 male, 76 female; mean age 41 years) from January 2002 to December 2021, with a confirmed diagnosis of PSC who underwent magnetic resonance imaging (MRI). Muscle mass was quantified at the level of the third lumbar vertebra by measurement of psoas muscle thickness (PMT) and total psoas muscle area (PMA). Sarcopenia was defined according to previously published cut‐off values. Muscle mass and the prevalence of sarcopenia were correlated with patient and disease characteristics, prognostic scoring systems (model for end‐stage liver disease (MELD) score; Mayo Risk Score; Amsterdam‐Oxford PSC Score) and clinical endpoints (liver transplantation, cirrhosis decompensation, liver‐related death).

**Results:**

Seventy‐eight patients reached a total of 104 clinical endpoints (liver transplantation *n* = 57, cirrhosis decompensation *n* = 28, liver‐related death *n* = 19). Sarcopenia was prevalent in 27.7% and 51.3%, respectively (according to the definition of PMT and PMA). Sarcopenia was significantly more prevalent in female patients and in patients without IBD (*p* < 0.05). A slight but significant negative correlation of muscle mass was noticed with the MELD (*r* = −0.244, *p* = 0.001) and Mayo Risk Score (*r* = −0.13, *p* = 0.046). At follow‐up, sarcopenia was associated with an inferior liver‐related event‐free survival (*p* < 0.05).

**Conclusions:**

Sarcopenia is highly prevalent in a large PSC cohort from a tertiary care centre, even more frequent in female patients and in patients without concomitant IBD. Furthermore, the presence of sarcopenia in PSC patients is associated with an inferior liver‐related event‐free survival.

**Abbreviations**
γ‐GT: γ‐glutamyltransferase
AIH: autoimmune hepatitis
ALT: alanine aminotransferase
AP: alkaline phosphatase
AST: aspartate aminotransferase
BSA: body surface area
CHE: cholinesterase
CT: computed tomography
DWI: diffusion‐weighted imaging
DXA: dual‐energy X‐ray absorptiometry
ERCP: endoscopic retrograde cholangiopancreatography
GRE: gradient echo
IBD: inflammatory bowel disease
ICC: intraclass correlation coefficient
INR: international normalised ratio
LFTs: liver function tests
MELD: Model for End‐Stage Liver Disease
MRCP: magnetic resonance cholangiopancreatography
MRI: magnetic resonance imaging
PMA: psoas muscle area
PMT: psoas muscle thickness
ROC: receiver operator characteristic
SC: primary sclerosing cholangitis
TIPS: transjugular intrahepatic portosystemic shunt

**Summary**

- Reduced muscle mass (sarcopenia) is a frequent finding in patients with primary sclerosing cholangitis, especially in female patients and in patients without concomitant inflammatory bowel disease.
- Patients affected have a higher probability of undergoing liver transplantation or experiencing cirrhosis decompensation.
- Muscle mass measurements can be performed easily using routine magnetic resonance imaging and have the potential to be useful in clinical trials, patients counselling and management.

## 1 Introduction

Primary sclerosing cholangitis (PSC) is a chronic cholestatic liver disease of unknown aetiology, characterised by diffuse fibrosing inflammation and obliteration of the intra‐ and extrahepatic bile ducts [1]. Recurrent cholangitis, biliary cirrhosis, and cholangiocarcinoma are typical complications, the latter with a 10‐year cumulative risk of 8% [2]. Even though the natural history of PSC is very variable, it slowly progresses to end‐stage liver disease in most patients [3]. The median survival rate from diagnosis to death or liver transplantation is 14.5 years [4]. Liver transplantation remains the only curative therapy; however, there is a risk of PSC recurrence of up to 20% [5].

Several prognostic models have been developed to predict life expectancy, including the Model for End‐Stage Liver Disease (MELD) Score for patients with chronic liver disease in general [6], and more specifically for PSC patients, the Mayo‐Risk Score [7] and Amsterdam‐Oxford model [8], respectively. While such prognostic models are useful to predict outcomes in patient cohorts, their ability to predict outcomes in an individual patient is limited and has even been recommended against [9]. Non‐invasive biomarkers that reflect PSC disease severity in the individual patient are still desperately needed for daily clinical practice and as stratification tools in clinical trials [1, 10]. In this sense, imaging‐based prognostic markers (e.g., ANALI score, DiStrict score, functional stricture) are increasingly evolving [11, 12, 13]. Their proof of suitability will have to be provided in the near future as they are currently not yet broadly applied.

Sarcopenia, which is defined as low muscle strength combined with a decline in muscle quantity and quality, is a condition that has been associated closely with chronic illnesses, deterioration of nutritional status, an impaired quality of life, and an increased risk for morbidity and mortality [14]. In this context, various imaging techniques can be employed to evaluate muscle mass, including dual‐energy X‐ray absorptiometry (DXA), computed tomography (CT) and magnetic resonance imaging (MRI).

More recently, the liver–muscle axis has gained increasing attention as it has become evident that the liver and skeletal muscles interact with each other [15] and sarcopenia has been shown to be highly prevalent in patients with chronic liver disease [16]. Specifically, in patients with cirrhosis as well as in patients undergoing liver transplantation, sarcopenia has been associated with a higher rate of complications and an inferior survival [16, 17, 18]. For example, in a recent meta‐analysis including 6965 patients, sarcopenia was associated with an approximately twofold higher risk of death in patients with cirrhosis, as compared to non‐cirrhotic patients [16]. Sarcopenia thus has emerged as a biomarker to predict clinical outcomes in patients with chronic liver disease. Despite this increasing evidence, data regarding the prevalence and significance of sarcopenia in patients with PSC specifically are very scarce and limited to small patient cohorts [19, 20, 21, 22].

MRI including magnetic resonance cholangiopancreatography (MRCP) is the imaging modality of choice for diagnosis and follow‐up of patients with PSC [1], making it an optimal tool for opportunistic evaluation of muscle mass. In addition, unlike DXA and CT, MRI enables quantification of muscle mass without the use of ionising radiation, making it a suitable technique for follow‐up measurements. The goal of our present study was twofold. First, to ascertain the prevalence of sarcopenia in a large cohort of PSC patients and second, to assess the prognostic value of muscle mass and sarcopenia as a prognostic biomarker for disease outcome.

## 2 Methods

### 2.1 Patients

This retrospective single‐centre study was approved by the Institutional Review Board with a waiver of patient consent granted. From January 2002 to December 2021, 290 patients with a confirmed diagnosis of PSC underwent routine MRI. The diagnosis of PSC was established as suggested by the European Association for the Study of the Liver: elevated serum markers of cholestasis not otherwise explained, characteristic cholangiographic bile duct changes with multifocal strictures and segmental dilatations, and causes of secondary sclerosing cholangitis and other cholestatic disorders excluded. For the diagnosis of cirrhosis, an extended definition was adopted. The diagnosis was thus either based on histology and/or imaging criteria (presence of liver dysmorphy with signs of portal hypertension, that is, platelet count < 150 × 10^9^/L, presence of oesophageal or gastric varices) [23]. Patients with small duct PSC, a subgroup characterised by a slowly progressive disease course and less frequent complications, were not included in this study. In each patient, the first available in‐house MRI scan was used for image analysis.

The authors reviewed electronic medical records including liver function tests (LFTs) closest to the time of the MRI. The MELD score (based on bilirubin, INR and serum creatinine) [6], Mayo risk score (based on patient age, serum levels of bilirubin, albumin, AST and history of variceal bleeding) [7] and the Amsterdam‐Oxford PSC model (based on PSC subtype, age at diagnosis, AST, AP, bilirubin, albumin and platelet count) [8] were calculated. Activity of concomitant inflammatory bowel disease (IBD), if present, was assessed clinically.

### 2.2 MRI and Image Analysis

**FIGURE 1:**
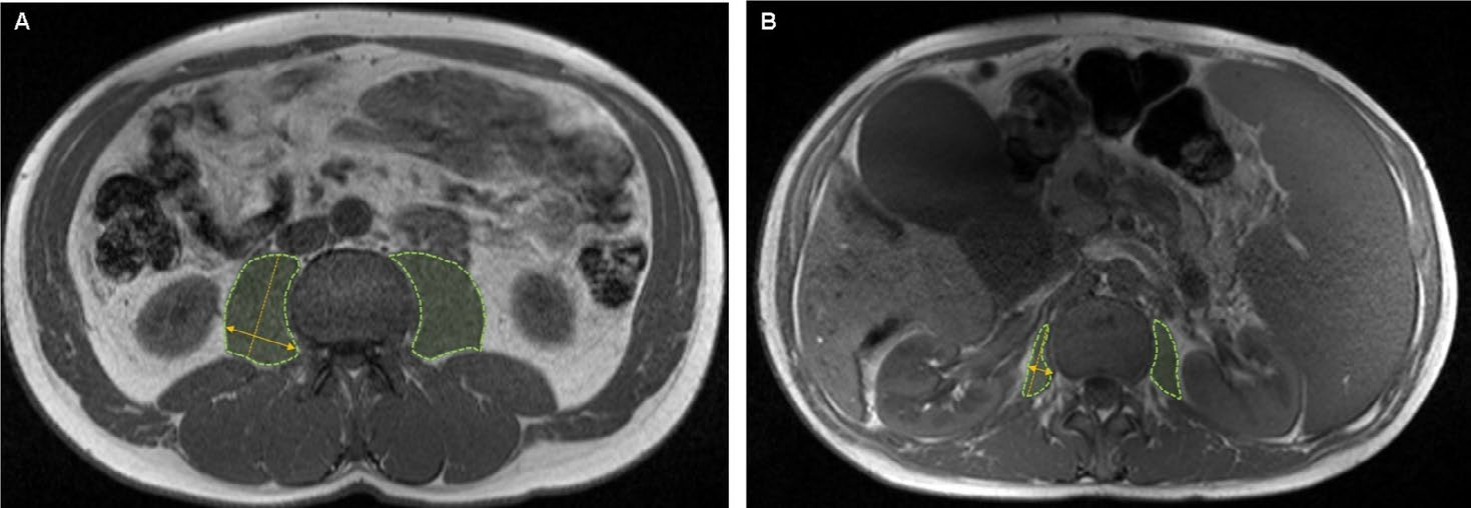
Muscle mass measurements were performed on a single slice at the level of the cranial endplate of the third lumbar vertebra. PMT: Psoas muscle thickness (yellow double arrow) was defined as the greatest diameter of the right psoas muscle perpendicular to the long axis (anterior–posterior oblique; yellow dashed line) and normalised to body height, as suggested by (20). PMA: Psoas muscle area (green area) was defined as the total muscle area of the right and left psoas muscle, as described previously by (15). (A) 37‐year‐old male PSC patient with PMT of 21.6 mm/m, PMA of 880 mm^2^ and uneventful follow‐up (7682 days). (B) 36‐year‐old male PSC patient with PMT of 4.9 mm/m and PMA of 563 mm^2^. 777 days after the MRI, the patient underwent liver transplantation.

MR images were acquired on a 1.5T (*n* = 202) or 3T (*n* = 22) scanner using phased‐array surface coils that covered the abdomen. The routine protocol consisted of non‐contrast and contrast‐enhanced T1‐weighted images, T2‐weighted images, diffusion‐weighted imaging (DWI) and a dedicated MRCP sequence. Image evaluation was performed on a commercially available workstation (Visage 7.1, Pro Medicus Inc., Melbourne, Australia), independently by one board‐certified radiologist (> 15 years of experience, reader 1) and one 5th year radiology resident (reader 2). Muscle mass was quantified on axial non‐contrast‐enhanced breath‐hold T1‐weighted 3D spoiled gradient echo (GRE) sequences with a slice thickness of 5 mm. Calculations were performed on a single slice at the level of the cranial endplate of the third lumbar vertebra (L3) in two different ways. First, the maximum transverse diameter of the right psoas muscle (PMT) was measured, and secondly, the total area of the psoas muscle on both sides (PMA) was calculated (Figure 1). Calculations were normalised to patient height, weight, and body surface area (BSA). Sarcopenia was defined according to previously published cut‐off values: for PMT < 8 mm/m in female and < 12 mm/m in male patients [24] and for PMA < 1464 mm [2] in female and < 1561 mm [2] in male patients [18], respectively.

### 2.3 Patient Follow‐Up

Routine patient follow‐up included LFTs and tumour markers (carcinoembryonic antigen, alpha‐fetoprotein, CA 19‐9) in 3‐ to 6‐month intervals, annual abdominal ultrasound (in case of liver cirrhosis every 6 months), MRI in 1‐ to 2‐year intervals (and in case of clinical deterioration), and ERCP in case of clinical signs of cholestasis, cholangitis, or detection of a relevant or high‐grade stricture at MRCP. The authors reviewed electronic medical records, especially concerning patient follow‐up. The following clinical endpoints were defined: patient undergoing liver transplantation, cirrhosis decompensation, or liver‐related death.

### 2.4 Statistical Analysis

Statistical analysis was performed using GraphPad Prism software (version 10; GraphPad Software Inc., USA) and SPSS version 22 (IBM, Armonk, NY). Patient demographic data is presented using descriptive statistics. Interrater agreement concerning the measurement of PMT and PMA was calculated using the intraclass correlation coefficient (ICC), which was interpreted as follows: a value less than 0.20 indicated poor agreement; a value of 0.21 to 0.40 fair agreement; a value of 0.41 to 0.60 moderate agreement; a value of 0.61 to 0.80 substantial agreement; and a value of 0.81 to 1.00 almost perfect agreement [25]. Muscle mass and the prevalence of sarcopenia were correlated with patient sex, concomitant IBD or autoimmune hepatitis (AIH) overlap syndrome (Mann–Whitney *U* test, Fisher‐exact test). Muscle parameters and sarcopenia were further correlated with LFTs, duration of disease, MELD score, Mayo‐Risk Score, Amsterdam‐Oxford model (Spearman correlation) and clinical endpoints (ROC‐analysis, logistic regression, Cox‐regression, Kaplan–Meier analysis). For all statistical analyses, a *p*‐value < 0.05 was considered significant.

## 3 Results

### 3.1 Study Population

Initially, 290 PSC patients were screened for study inclusion. Exclusion criteria were as follows: patient age < 18 years (*n* = 3), diagnosis of small duct PSC (*n* = 7), established diagnosis of cholangiocarcinoma prior to or at the time of the respective MRI (*n* = 8), status post liver transplantation (*n* = 46) or transjugular intrahepatic portosystemic shunt (TIPS; *n* = 2) at the time of imaging. Finally, a total of 224 patients (148 male, 76 female; mean age 41 years; range 18–67 years) were included in this study. Concomitant IBD was present in 70.1%. The majority (82%) had a diagnosis of ulcerative colitis. At the time of MRI for evaluation of sarcopenia, 14% had undergone IBD‐related surgery for management of their disease and 70% were on medication (5‐ASA, *n* = 92; immunomodulators *n* = 23; biological agents *n* = 8, steroid therapy *n* = 24). Clinically, the majority of patients were in remission (81%); 10.8% had moderate IBD‐related symptoms and 8.2% suffered from active disease with recurrent flares.

Our patient cohort was characterised by predominantly moderate disease stage and intermediate risk, as reflected by a mean MELD score and Mayo Risk Score of 9 and 1.9, respectively. Sixty‐eight patients (30.4%) had a diagnosis of cirrhosis, and 60 patients (26.8%) had a history of acute cholangitis. A dominant stricture at ERCP was present in 65 patients (29%), with a total of 95 patients (42%) having undergone endoscopic intervention prior to the time of imaging. Patient demographic data is presented in detail in Table 1.

**TABLE 1:** Patient cohort characteristics.

Patients	224
Male/female	148/76
Age (years)	41 (31–50)
Age at PSC diagnosis (years)	34 (23–42)
PSC‐AIH overlap	38 (17)
Concomitant IBD	157 (70)
Ulcerative colitis	129 (82.2)
Crohn's disease	23 (14.6)
Indeterminate colitis	5 (3.2)
Diagnosis of cirrhosis^a^	68 (43)
Based on histology	58 (85.3)
Based on imaging	34 (50)
MELD score	9 (7–11)
Mayo Risk Score	1.9 (0.08–3.51)
Amsterdam Oxford model (*n* = 55)	2.06 (1.59–2.46)
Follow‐up (months)	77 (26–120)
Endpoints (*n*)	104 (in 78 patients)
Cirrhosis decompensation	28
Liver transplantation	57
Liver‐related death	19

*Note:* Values are *n* (%) or mean (interquartile range).

^a^ Patients with histologically confirmed cirrhosis and/or diagnosis based on imaging criteria.

### 3.2 Evaluation of Muscle Mass and Presence of Sarcopenia

PMT and PMA could be measured effectively in all patients. Interrater agreement was almost perfect for both parameters as reflected by an ICC of 0.91 (95% confidence interval 0.76–0.93) for PMT and 0.96 (95% confidence interval 0.89–0.98) for PMA, respectively. For further subgroup analysis and correlations with clinical data, measurements performed by reader 1 were used. Using these measurements, two different approaches for the definition of sarcopenia were applied, based either on PMT normalised to patient height or based on PMA. Respective cut‐off values are provided in the materials and methods section of this manuscript.

PMT and PMA correlated significantly with patient weight, height, and Body Mass Index (BMI) (all *p* < 0.005).

Sarcopenia, as defined by PMT and PMA, was present in 27.7% and 51.3% of the total patient cohort, respectively (Table 2). Sarcopenic patients had a significantly lower BMI. PMT and PMA measurements were significantly lower in female patients (*p* < 0.0001 for both). In this context, sarcopenia as defined by PMA only was significantly more prevalent in the female population as compared to males (81.6% vs. 35.8%; *p* < 0.0001). At subgroup analysis, absolute measurements of psoas muscle thickness (PMT) and psoas muscle area (PMA) were significantly higher in patients with concomitant IBD (*p* = 0.04 and *p* = 0.003). Consequently, sarcopenia was significantly more demonstrable in patients without IBD (as defined by PMA: 64.2% vs. 45.8%; *p* < 0.01). At further analysis, sarcopenia (as defined by PMT) was less prevalent in patients with IBD in remission status, as compared to patients with moderate symptoms or active disease with recurrent flares (*p* = 0.014).

**TABLE 2:** Demographic data of PSC patients included in this study and characteristics stratified by muscle measurements and presence of sarcopenia.

Parameter		PMT (mm/m)	PMA (mm^2^)	Sarcopenia (defined by PMT)	Sarcopenia (defined by PMA)
General patient data					
Total	*n* = 224	12.7 (10.3–15.2)	1577 (1103–2080)	*n* = 62 (27.7%)	*n* = 115 (51.3%)
Sex					
Male	*n* = 148	13.9 (11.5–16.5)	1827 (1434–2283)	*n* = 44 (29.7%)	*n* = 53 (35.8%)
Female	*n* = 76	10.4 (8.1–12.5) *p* < 0.0001^a^	1089 (790–1286) *p* < 0.0001^a^	*n* = 18 (23.7%) *p* = 0.213	*n* = 62 (81.6%) *p* < 0.0001^a^
Age (years)	41 (31–50)	*r* = −0.076 *p* = 0.251	*r* = −0–046 *p* = 0.474	Yes: 42 (31–54) No: 40 (32–50) *p* = 0.227	Yes: 41 (31–52) No: 41 (33–50) *p* = 0.49
Baseline clinical data					
Cirrhosis					
Yes	*n* = 68 (30.4%)	11.6 (8.1–14.1)	1394 (900–1809)	*n* = 27	*n* = 43
No	*n* = 156 (69.6%)	13.3 (11.1–15.5) *p* = 0.0031^a^	1657 (1162–2102) *p* = 0.0015^a^	*n* = 41 *p* = 0.007^a^	*n* = 72 *p* = 0.013^a^
AIH overlap					
Yes	*n* = 38 (17%)	13 (10–15.9)	1562 (1011–2137)	*n* = 6 (15.8%)	*n* = 19 (50.2%)
No	*n* = 186 (83%)	12.7 (10.4–15) *p* = 0.473	1580 (1112–2043) *p* = 0.422	*n* = 56 (30.1%) *p* = 0.05	*n* = 96 (51.6%) *p* = 0.498
IBD					
Yes^b^	*n* = 157 (70%)	13 (10.8–15.8)	1647 (1160–2104)	*n* = 40 (25.5%)	*n* = 72 (45.9%)
No	*n* = 67 (30%)	12.1 (9.4–14.9) *p* = 0.04^a^	1414 (928–1705) *p* = 0.003^a^	*n* = 22 (32.8%) *p* = 0.167	*n* = 43 (64.2%) *p* = 0.008^a^
Baseline LFTs^c^					
AST (U/L)	69 (32–89)	*r* = −0.111 *p* = 0.068	*r* = −0.114 *p* = 0.063	Yes: 76 (47–98) No: 67 (32–87) *p* = 0.008^a^	Yes: 73 (36–97) No: 66 (32–84) *p* = 0.068
ALT (U/L)	84 (31–105)	*r* = 0.044 *p* = 0.276	*r* = 0.057 *p* = 0.223	Yes: 84 (36–115) No: 87 (31–103) *p* = 0.349	Yes: 87 (32–110) No: 86 (32–105) *p* = 0.453
AP (U/L)	273 (127–396)	*r* = −0.239 *p* < 0.001^a^	*r* = −0.48 *p* = 0.025^a^	Yes: 394 (193–567) No: 241 (120–313) *p* < 0.001^a^	Yes: 315 (146–449) No: 249 (124–321) *p* = 0.029^a^
Bilirubin (μmol/L)	40 (10–34)	*r* = −0.132 *p* = 0.042^a^	*r* = −0.132 *p* = 0.069	Yes: 68 (12–84) No: 3 (10–27) *p* < 0.001^a^	Yes: 47 (10–42) No: 38 (10–24) *p* = 0.051^a^
γ‐GT (U/L)	241 (62–333)	*r* = −0.038 *p* = 0.305	*r* = −0.114 *p* = 0.458	Yes: 270 (95–427) No: 241 (58–309) *p* = 0.012^a^	Yes: 248 (72–363) No: 251 (68–343) *p* = 0.26
CHE (kU/L)	11.1 (4.8–7.9)	*r* = 0.35 *p* < 0.0001^a^	*r* = 0.292 *p* < 0.0001^a^	Yes: 4.9 (2.8–6.5) No: 8.6 (5.2–8.1) *p* < 0.001^a^	Yes: 5.6 (3.5–7.6) No: 9.8 (5.5–8.4) *p* < 0.001^a^
Albumin (g/L)	37 (33–42)	*r* = 0.289 *p* = 0.003^a^	*r* = 0.327 *p* = 0.001^a^	Yes: 32 (27–38) No: 38 (35–42) *p* = 0.004^a^	Yes: 35 (30–41) No: 39 (36–44) *p* = 0.002^a^
Clinical scores					
MELD (*n* = 176 patients)	9 (7–11)	*r* = −0.244 *p* = 0.001^a^	*r* = −0.205 *p* = 0.005^a^	yes: 13 (8–16) no: 8 (6–9) *p* < 0.0001^a^	yes: 11 (7–13) no: 8 (6–9) *p* = 0.009^a^
Mayo risk (*n* = 193 patients)	1.9 (0.1–3.5)	*r* = −0.13 *p* = 0.046^a^	*r* = −0.146 *p* = 0.029^a^	Yes: 2.6 (1.2–4) No: 1.7 (−0.2–3.6) *p* = 0.0024^a^	Yes: 2.1 (0.6–3.6) No: 1.8 (−0.2–3.6) *p* = 0.238
Amsterdam‐Oxford model (*n* = 55 patients)	2 (1.6–2.5)	*r* = −0.190 *p* = 0.102	*r* = −0.222 *p* = 0.069	Yes: 2.5 (1.7–3.3) No: 1.9 (1.5–2.4) *p* = 0.045^a^	Yes: 2.2 (1.7–2.6) No: 1.9 (1.4–2.4) *p* = 0.176

*Note:* Values are presented as mean with interquartile range in brackets, if not stated otherwise. Sarcopenia was defined either on psoas muscle thickness (PMT) or psoas muscle area (PMA) based on previously published cut‐off values.

Abbreviations: CCA = cholangiocarcinoma; n.a. = not assessed; Tx = transplantation.

^a^ Statistical relevance.

^b^ 129 patients with ulcerative colitis, 23 patients with Crohn's disease and 5 patients with indeterminate colitis.

^c^ 184 patients with LFTs available within 1 week of MRI.

Similarly, sarcopenia was significantly more present in patients with a history of an acute cholangitis episode (PMT: *p* = 0.0006; PMA: *p* = 0.0098) and in patients with cirrhosis (PMT: *p* = 0.007; PMA: *p* = 0.013). Regarding muscle mass measurements and the presence of sarcopenia, there were no significant differences between subgroups looking at concomitant AIH‐overlap syndrome (yes vs. no), the presence of a dominant stricture (yes vs. no) or previous endoscopic intervention (yes vs. no). No correlations between muscle mass and disease duration prior to MRI or age at PSC diagnosis were observed (*p* > 0.05). At univariable analysis, the presence of cirrhosis, a history of acute cholangitis, female sex, the presence of IBD, and active IBD were significant risk factors for sarcopenia as defined by PMA and/or PMT. At subsequent multivariable analysis, the diagnosis of cirrhosis, a history of an acute cholangitis episode, female sex, and the presence of active IBD remained significant risk factors for the development of sarcopenia (Tables 3 and 4).

**TABLE 3:** Logistic regression analysis of risk factors for development of sarcopenia as defined by psoas muscle area (PMA) in the whole cohort.

	Univariable		Multivariable	
	*p*	OR (95% CI)	*p*	OR (95% CI)
Presence of cirrhosis	0.0196^a^	2.007 (1.125–3.637)	0.0627	1.872 (0.967–3.664)
Presence of dominant stricture	0.4583	0.814 (0.4554–1.451)		
Previous bile duct intervention	0.1586	1.468 (0.863–2.511)		
History of acute cholangitis	0.0014^a^	2.150 (1.175–4.022)	0.0259^a^	2.182 (1.098–4.417)
Female sex	< 0.0001^a^	7.938 (4.162–16.02)	< 0.0001^a^	8.206 (4.202–16.98)
Presence of IBD	0.0128^a^	0.4728 (0.259–0.847)	0.1334	0.598 (0.303–1.170)
Active IBD	0.3061	1.5 (0.6918–3.356)		

Abbreviations: CI = confidence interval; IBD = inflammatory bowel disease; OR = odds ratio.

^a^ Statistical significance.

**TABLE 4:** Logistic regression analysis of risk factors for development of sarcopenia as defined by psoas muscle thickness (PMT) in the whole cohort.

	Univariable		Multivariable	
	*p*	OR (95% CI)	*p*	OR (95% CI)
Presence of cirrhosis	0.0087^a^	2.277 (1.229–4.215)	0.015^a^	6.549 (1.223–4.523)
Presence of dominant stricture	0.509	1.238 (0.649–2.318)		
Previous bile duct intervention	0.1563	1.531 (0.849–2.767)		
History of acute cholangitis	0.0006^a^	3.016 (1.604–5.689)	0.0012^a^	10.43 (1.532–5.687)
Female sex	0.3343	0.7335 (0.3818–1.369)		
Presence of IBD	0.2609	0.6993 (0.376–1.316)		
Active IBD	0.0472^a^	2.263 (1.011–4.982)	0.0127^a^	2.967 (1.267–6.909)

Abbreviations: CI = confidence interval; IBD = inflammatory bowel disease; OR = odds ratio.

^a^ Statistical significance.

### 3.3 Correlation of Muscle Mass With Liver Function and Clinical Scores

Liver function in 184 patients with LFTs available within 1 week of the MRI and respective muscle mass measurements was analysed. Significant correlations of several LFTs with muscle mass and presence of sarcopenia were observed, including especially cholinesterase (*r* = 0.35), albumin (*r* = 0.289) and creatinine (*r* = 0.342; all *p* < 0.01) (Table 2). A slight but also significant negative correlation of muscle mass was further noticed with the MELD (*r* = −0.244, *p* = 0.001) and Mayo Risk Score (*r* = −0.13, *p* = 0.04). Accordingly, sarcopenic patients had significantly higher scores as compared to non‐sarcopenic patients (Table 2).

### 3.4 Patient Follow‐Up and Correlation of Muscle Mass With Clinical Endpoints

Median follow‐up after MR imaging was 73.5 months (interquartile range 26–120 months). During this follow‐up period, 104 clinical endpoints were observed in 78 patients (34.8%). This includes 24 patients with two different endpoints and one patient with three different endpoints. Specifically, 57 patients underwent liver transplantation, 28 patients developed cirrhosis decompensation and liver‐related death occurred in 19 patients. Median interval to first event from MRI was 22.5 months. Even though sarcopenia was slightly less prevalent in patients not developing endpoints, ROC and regression analysis did not reveal a significant correlation of muscle mass with endpoints, neither for the composite endpoint nor for individual endpoints. The most frequent endpoint observed in our study was liver transplantation (*n* = 57). Looking therefore in more detail at patients undergoing liver transplantation, at univariable analysis, diagnosis of cirrhosis, cirrhosis decompensation, presence of a dominant stricture, previous bile duct interventions, and history of acute cholangitis were significant risk factors for liver transplantation (all *p* < 0.05). At subsequent multivariable analysis, only diagnosis of cirrhosis and history of acute cholangitis remained significant (*p* = 0.0003 and 0.0452; OR = 3.559 and 2.101).

**FIGURE 2:**
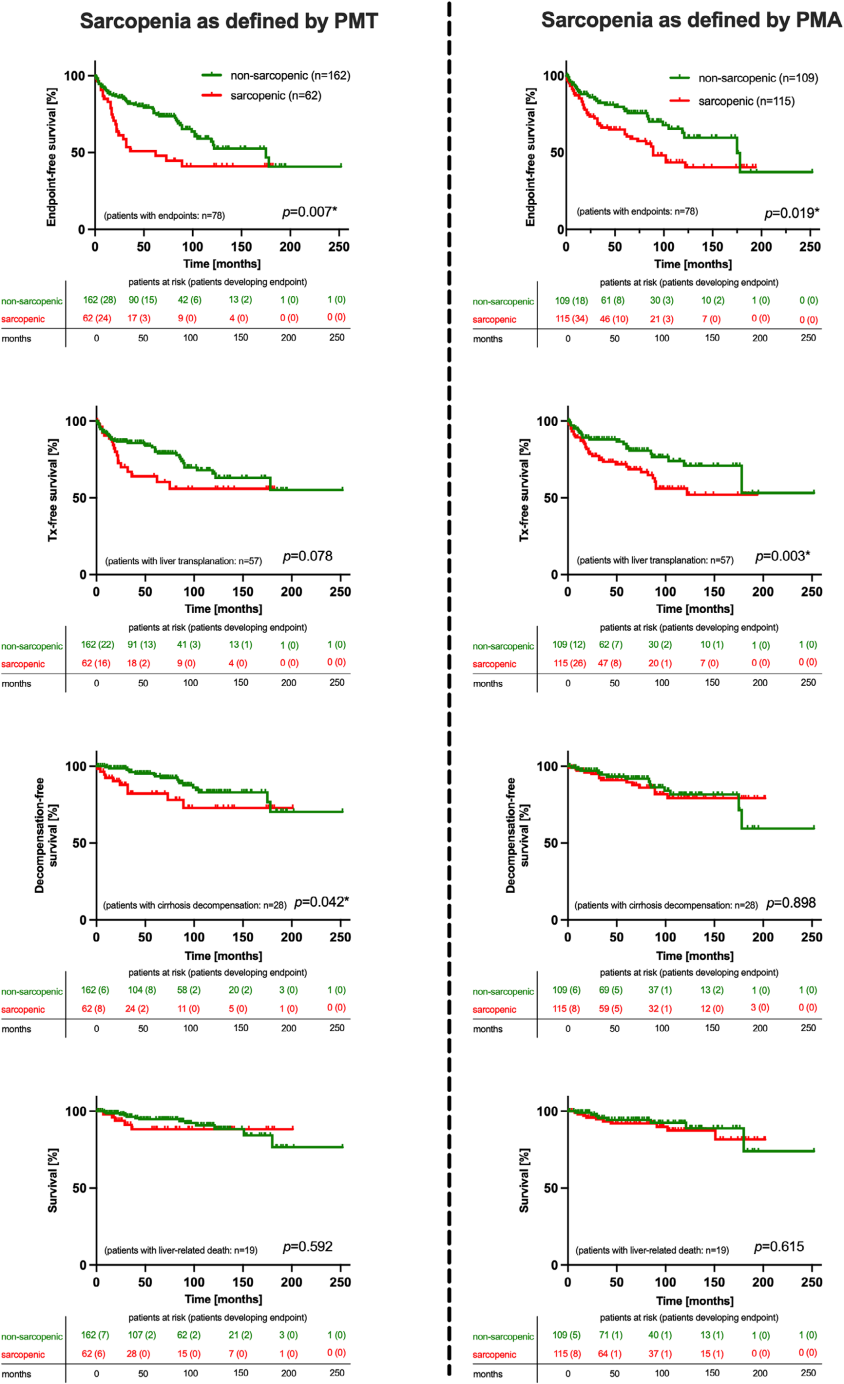
Kaplan–Meier curves of event‐free survival in patients with PSC, risk‐stratified based on presence of sarcopenia or not. PMA = psoas muscle area; PMT = psoas muscle thickness; tx = transplantation. *Statistical significance (log‐rank test).

**FIGURE 3:**
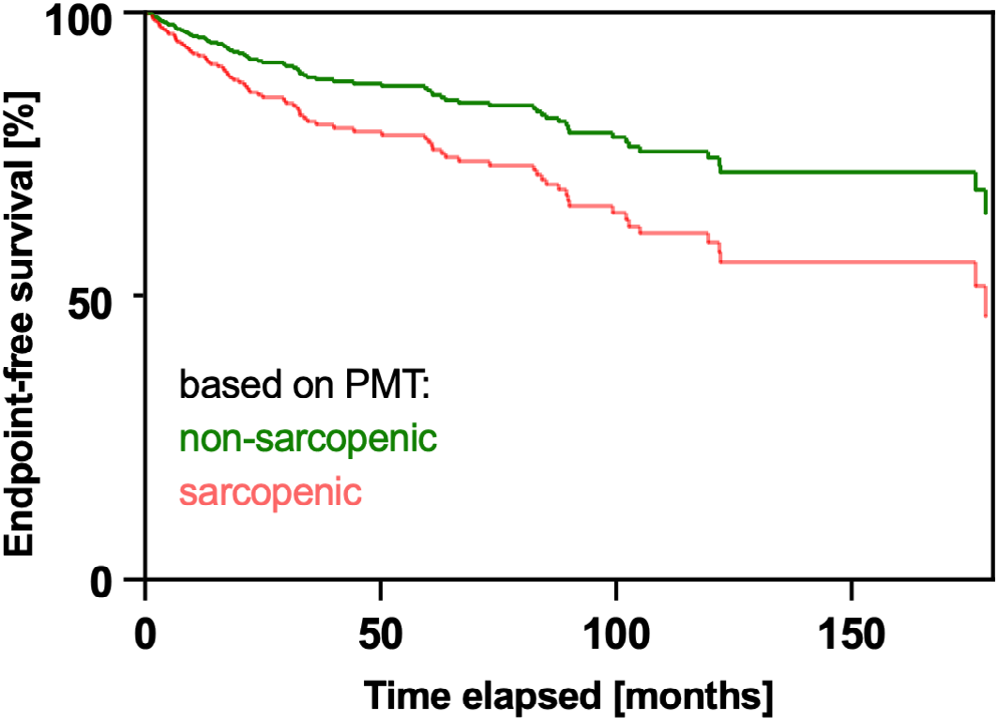
Cox proportional hazards regression of endpoint‐free survival in patients with PSC, stratified by the presence of sarcopenia as defined by psoas muscle thickness (PMT).

At Kaplan–Meier analysis, event‐free survival (composite endpoint), transplant‐free survival, and cirrhosis decompensation‐free survival were also significantly higher in non‐sarcopenic patients (all *p* < 0.05; Figure 2). Multivariable Cox proportional hazards regression was performed for prediction of the composite endpoint (liver‐related events: transplantation, cirrhosis decompensation, liver‐related death). For analysis, the first available event was considered. In this sense, the diagnosis of cirrhosis and the presence of sarcopenia as defined by PMT were significant contributing factors (*p* < 0.0001 and 0.0411; HR 3.446 and 1.753; Table 5, Figure 3).

**TABLE 5:** Multivariable Cox proportional hazards regression for prediction of liver‐related events.

	*p*	HR	95% CI
Diagnosis of cirrhosis^a^	< 0.0001^c^	3.446	2.116–5.597
Sarcopenia as defined by PMA	0.3457	1.272	0.7635–2.126
Sarcopenia as defined by PMT	0.0411^c^	1.753	1.023–2.971
Presence of IBD	0.1378	1.448	0.8895–2.402
Time (PSC diagnosis to MRI)^b^	0.3673	1	0.9998–1

*Note:* Liver‐related events refer to the composite endpoint (including liver‐related death, liver transplantation and decompensation of cirrhosis). For analysis, the first available event was considered.

Abbreviations: CI = confidence interval; HR = hazard ratio; PMA = psoas muscle area; PMT = psoas muscle thickness.

^a^ Patients with diagnosis based either on histology and/or imaging criteria.

^b^ Time interval between PSC diagnosis and MRI for sarcopenia evaluation.

^c^ Statistical relevance.

## 4 Discussion

Previous studies have shown that the prevalence of sarcopenia in patients with chronic liver disease in general is high, and the prevalence of sarcopenia has been shown to be associated with an increased likelihood of adverse outcomes. PSC is a chronic liver disease slowly progressing to end‐stage liver disease in most patients. The association of muscle mass and the prognostic value of sarcopenia in patients with PSC, however, has yet to be determined. In the present study, we demonstrated that sarcopenia is highly prevalent (up to 51%) in a large PSC cohort from a tertiary referral centre. Furthermore, the prevalence of sarcopenia was significantly higher in female patients as well as in patients without concomitant IBD. A weak but significant negative correlation of muscle mass was observed with the MELD and Mayo‐Risk Score, respectively. Even though muscle mass was higher in patients with uneventful follow‐up, at Kaplan–Meier analysis, sarcopenia was significantly associated with inferior transplant‐free survival and decompensation‐free survival. Cox‐regression analysis identified sarcopenia (as defined by PMT) as a significant contributing factor with regard to liver‐related event‐free survival (composite endpoint), attesting sarcopenia a potential predictive role in patients with PSC.

In our study, multivariable logistic regression analysis identified cirrhosis and history of acute cholangitis as significant risk factors for liver transplantation. Cirrhosis is in line with the natural course of patients with PSC. Of note, acute cholangitis (and similarly the loss of body weight of at least 10% within 12 months) has been standard exception criteria in Germany for admitting patients to the transplant waiting list up to September 2023. Interestingly, we found that a history of acute cholangitis was also an independent risk factor for liver transplantation in PSC, even though the presence of a dominant stricture was not. Therefore, it appears that acute cholangitis (with its systemic effects and weight loss episodes) has a more direct influence on sarcopenia, whereas the mere presence of a stricture does not have such an impact. The recently introduced ‘relevant stricture’ framework (associated with symptoms like cholangitis or biochemical deterioration) could bring further clarification in this context [1]. Due to the retrospective nature of this study, we can unfortunately not provide a precise rate of hospitalisation among the 60 patients with a history of acute cholangitis. Based on clinical practice and guidelines, it is likely that the majority of episodes were treated in hospital. Hospitalisation for acute cholangitis itself implies a more severe degree, and at least in theory, the longer the episode the more the potential effect on malnutrition and development of sarcopenia can be assumed. However, we do not consider hospitalisation per se to be the cause of sarcopenia. Rather, the systemic inflammatory response and catabolic stress associated with acute cholangitis are more plausible drivers of muscle loss.

It has been suggested that sarcopenia is a better objective measure of denutrition in patients with cirrhosis as compared to serum (pre‐) albumin, patient weight, or BMI, which may be modified by hepatopathy or ascites [18]. However, heterogeneity exists in the literature with respect to the site and specific type of measurements for imaging‐based assessment of sarcopenia. In our present study, we considered two different approaches for measurements of muscle mass, namely PMT normalised to body height and total PMA. Based on previously published cut‐off values for each method, the prevalence of sarcopenia was distinctly different (PMT‐based: 28% vs. PMA‐based: 51%, respectively). Interrater agreement was almost perfect for both approaches, but slightly higher for PMA measurements. Even though both methods are valid, from our experience, PMA might be the preferable approach as correlations with clinical parameters were more evident. Also, measurement of muscle area may be less susceptible to variations as it is not dependent on muscle shape. At least in theory, deformation of the psoas muscle can occur with muscle contraction, and it could vary with exact body positioning, thus having an impact on muscle thickness measurements. Furthermore, it has been suggested that PMA may be affected less by ascites or hepatomegaly [18, 26]. Respective cut‐off values for the definition of sarcopenia, however, should be further validated in a multicentre setting [1].

Few previous studies have evaluated the significance of sarcopenia specifically in patients with PSC. Shteyer et al. calculated the psoas muscle surface area in 20 patients younger than 25 years and in 45 controls [19]. Preliminary results so far are published only as a short abstract; therefore, the specific methodology unfortunately cannot be reconstructed in detail. PMA correlated with AST and the APRI (aspartate aminotransferase to platelet ration index). Even though mean patient age was distinctly younger (mean age 15 years) as compared to our adult PSC cohort (mean age 41 years), in line with our results, PMA was significantly higher in patients with concomitant IBD. Recently, it has been suggested that the cholestasis‐associated malabsorption in patients with PSC may be worsened by eventual concomitant IBD [27]. However, as demonstrated by Shteyer et al. and confirmed by our study, the contrary seems to be the case. A recent review on osteosarcopenia in autoimmune liver disease denoted this as a ‘curious finding’ that should be confirmed in larger studies [27]. In our present study, patients with IBD presented with a better muscle status, suggesting that nutrition regimens in this specific subpopulation may have a rather protective effect. Interestingly, at subgroup analysis, sarcopenia was less prevalent in patients with IBD in remission status as compared to patients with active IBD. In this sense, active IBD was a risk for the development of sarcopenia as defined by PMT. These results suggest that IBD activity, rather than mere presence, has a relevant role in sarcopenia development. Nevertheless, it is important to keep in mind that sarcopenia specifically in IBD cohorts has a prevalence of 27% to 61% and was shown to be a negative prognostic factor for intestinal resection [28].

Kikuchi et al. performed CT‐based measurements in 22 patients with PSC and 44 matched controls, including calculation of the psoas muscle index (PMA normalised to patient height squared) [21]. Significant correlations between PMI, the MELD score, liver transplantation, and death were observed only in men, implying the presence of gender‐specific differences. In contrast to our study, the patient population of Kikuchi and colleagues was much smaller (22 vs. 231 patients) with events in only three patients.

In line with our present results, Seifert et al. identified sarcopenia as an independent risk factor for transplant‐free survival at 2 and 5 years following cross‐sectional imaging in 91 patients with PSC [20]. Unfortunately, data has been presented so far only in the form of an abstract, and further information regarding exact methodology and follow‐up is not available in detail yet. More recently, an interesting study was published evaluating not muscle mass but rather muscle quality in terms of intramuscular fat fraction (IMFF) as a surrogate for myosteatosis [22]. In 116 PSC patients, IMFF was significantly associated with survival, and at multivariate analysis, an IMFF ≥ 15% was identified as an independent predictor for transplant‐free survival. There was no difference in IMFF regarding concomitant IBD. The results of that study suggest that an increased fat infiltration of skeletal muscle is associated with a more advanced disease stage and worse outcome [22]. Methodologically, quantification of muscle quality is a little bit more cumbersome as compared with simple muscle mass measurements. In the above‐mentioned study by Praktiknjo et al., the cross‐sectional area of the paraspinal muscle compartment, including the erector spinae muscles and the spinotransversal muscle group, was manually traced. An in‐house tool was then used for quantification of muscle fat infiltration. Though very interesting, this approach may be less practical to adopt in clinical routine for opportunistic screening.

In comparison, the technique of muscle mass measurements used in our study can be easily incorporated in clinical routine. It is simple, very robust and applicable for general use in multicenter trials as no dedicated software or expertise is necessary. The clinical workflow of the proposed method could be further enhanced by integrating deep learning‐based algorithms for the segmentation of the psoas muscle, which has already been suggested for abdominal CT scans [29]. Another major strength of our study is the large PSC cohort itself, with a comparable long follow‐up and a considerate number of endpoints, given the natural course of PSC. Our study does have some limitations. This is a retrospective single‐centre study and applied cut‐off values should be validated in a multicenter setting. As a tertiary referral centre, our cohort reflects a potential selection bias towards more severe disease. Second, mean disease duration up to MRI and evaluation of sarcopenia was rather long (mean 7.3 years). We are currently planning a longitudinal study in order to evaluate intra‐individual changes of muscle mass over time in correlation with the disease course. Third, we did not assess muscle quality (myosteatosis) but only muscle mass, which seems more practical in daily routine. Last, we acknowledge that immunosuppressive treatment is a potential confounder in PSC‐AIH overlap patients, which should be considered in future studies.

In conclusion, the results of our study reveal a high prevalence of sarcopenia in patients with PSC. Male sex and concomitant IBD seem rather protective factors. As a prognostic biomarker concerning disease progression, sarcopenia is associated with an inferior liver‐related event‐free survival. Screening for sarcopenia in PSC patients undergoing MRI, therefore, is convenient and easy to implement. More importantly, routine assessment may be beneficial for risk stratification and early identification of individuals, enabling early initiation of specific counteractive (e.g., nutritional) measures.

## Author Contributions

**Alena Levers:** conception and design of work, acquisition, interpretation of data, drafting of work, final approval of the version to be published, agreement to be accountable for all aspects of the work. **Judith Pantke:** acquisition, interpretation of data, critical revising of work, final approval of the version to be published, agreement to be accountable for all aspects of the work. **Filip Klimeš:** acquisition, analysis, interpretation of data, critical revision of work, final approval of the version to be published, agreement to be accountable for all aspects of the work. **Henrike Lenzen:** acquisition, interpretation of data, critical revising of work, final approval of the version to be published, agreement to be accountable for all aspects of the work. **Daniel Düx:** acquisition, interpretation of data, critical revising of work, final approval of the version to be published, agreement to be accountable for all aspects of the work. **Richard Taubert:** acquisition, analysis, interpretation of data, critical revising of work, final approval of the version to be published, agreement to be accountable for all aspects of the work. **Heiner Wedemeyer:** acquisition, interpretation of data, critical revising of work, final approval of the version to be published, agreement to be accountable for all aspects of the work. **Frank Wacker:** conception, acquisition, interpretation of data, critical revising of work, final approval of the version to be published, agreement to be accountable for all aspects of the work. **Kristina I. Ringe:** conception and design of work, analysis and interpretation of data, drafting of work, final approval of the version to be published, agreement to be accountable for all aspects of the work.

## Ethics Statement

This study was approved by the institutional review board of Hannover Medical School.

## Consent

Patient consent was waived by the institutional review board for this retrospective study.

## Conflicts of Interest

The authors declare no conflicts of interest.

## Data Availability

The data that support the findings of this study are available from the corresponding author upon reasonable request.
